# mTOR Signaling Pathway in Bone Diseases Associated with Hyperglycemia

**DOI:** 10.3390/ijms24119198

**Published:** 2023-05-24

**Authors:** Shuangcheng Wang, Jiale Wang, Shuangwen Wang, Ran Tao, Jianru Yi, Miao Chen, Zhihe Zhao

**Affiliations:** 1 State Key Laboratory of Oral Diseases, National Clinical Research Center for Oral Diseases, West China Hospital of Stomatology, Sichuan University, Chengdu 610041, China; 2 West China School of Medicine, Sichuan University, Chengdu 610041, China; 3 Department of Orthodontics, West China Hospital of Stomatology, Sichuan University, Chengdu 610041, China

**Keywords:** mTOR signaling, bone complications, diabetes

## Abstract

The interplay between bone and glucose metabolism has highlighted hyperglycemia as a potential risk factor for bone diseases. With the increasing prevalence of diabetes mellitus worldwide and its subsequent socioeconomic burden, there is a pressing need to develop a better understanding of the molecular mechanisms involved in hyperglycemia-mediated bone metabolism. The mammalian target of rapamycin (mTOR) is a serine/threonine protein kinase that senses extracellular and intracellular signals to regulate numerous biological processes, including cell growth, proliferation, and differentiation. As mounting evidence suggests the involvement of mTOR in diabetic bone disease, we provide a comprehensive review of its effects on bone diseases associated with hyperglycemia. This review summarizes key findings from basic and clinical studies regarding mTOR’s roles in regulating bone formation, bone resorption, inflammatory responses, and bone vascularity in hyperglycemia. It also provides valuable insights into future research directions aimed at developing mTOR-targeted therapies for combating diabetic bone diseases.

## 1. Introduction

Diabetes mellitus (DM) is a metabolic disorder that has become a worldwide epidemic [1]. It is characterized by hyperglycemia and multisystem complications that can impact patients’ quality of life and impose a significant socioeconomic burden on individuals and society [2]. The most well-documented DM complications include microvascular complications, such as retinopathy and nephropathy, and macrovascular complications, such as cardiovascular disease [3]. In recent decades, it has been increasingly recognized that DM also impairs bone health. Individuals with type 1 diabetes mellitus (T1DM) experience insulinopenia, which attenuates bone anabolism and results in reduced bone mineral density (BMD) [4], and an approximately sevenfold increase in the risk of hip fracture [5]. On the other hand, type 2 diabetes mellitus (T2DM) is associated with normal or high BMD but paradoxically increased fracture risk due to hyperglycemia-induced alterations in organic ingredients and skeletal microarchitecture [6]. Additionally, diabetic patients experience prolonged fracture healing times of approximately 87%, with a higher risk of delayed union, redislocation, and pseudoarthrosis [7,8,9]. With the increasing incidence of DM and the substantial socioeconomic burden it imposes globally, there is a pressing need for an improved understanding of bone metabolism in hyperglycemia.

The mammalian target of rapamycin (mTOR) is an evolutionarily conserved serine/threonine kinase that acts as a central regulator of cellular and organismal growth and homeostasis [10,11]. It integrates various environmental inputs from nutrients and growth factors to regulate a diverse array of physiological processes, including macromolecular synthesis, ribosome biogenesis, cell growth, survival, and autophagy [12]. Initially, mTOR is considered a target of interest in cancer control due to its proliferation control. Later, mounting evidence confirmed that mTOR is particularly important to metabolic balance as well due to its response to nutrients [13]. Indeed, various endocrine disorders including DM and insulin resistance which are induced by aberrant energy homeostasis are often accompanied by deregulated mTOR signaling [10,11,14]. Hence, mTOR has been proposed as a promising therapeutic target for DM treatment [10,13]. Moreover, mTOR dysregulation is also heavily implicated in diabetes-related complications, including nephropathy, heart failure, neuropathy, and diabetic osteoporosis [4,15,16,17]. Regarding bone health, mTOR signaling is crucial to multiple aspects of skeletal development and health. Furthermore, mTOR signaling is crucial to multiple aspects of skeletal development and health [18]. Dysregulation of mTOR pathways renders bone marrow mesenchymal stem cells (BMSCs) unable to proliferate and differentiate properly, leading to bone loss and osteoporosis [18,19,20,21]. Recently, mTOR signaling has emerged as a pivotal regulator in bone metabolism under hyperglycemic conditions [22], making targeting mTOR a plausible approach for treating diabetic bone disorders. Thus, understanding the detailed molecular process of mTOR-regulated bone metabolism in hyperglycemia is critical for developing strategies to combat diabetic bone diseases. Herein, we summarized the current knowledge of mTOR in bone metabolism, with a particular emphasis on its role in diabetic bone disorders and the therapeutic potential of targeting mTOR pathways for bone health in diabetic patients.

## 2. mTOR Signaling Pathways

### 2.1. mTOR Complexes

**Figure 1:**
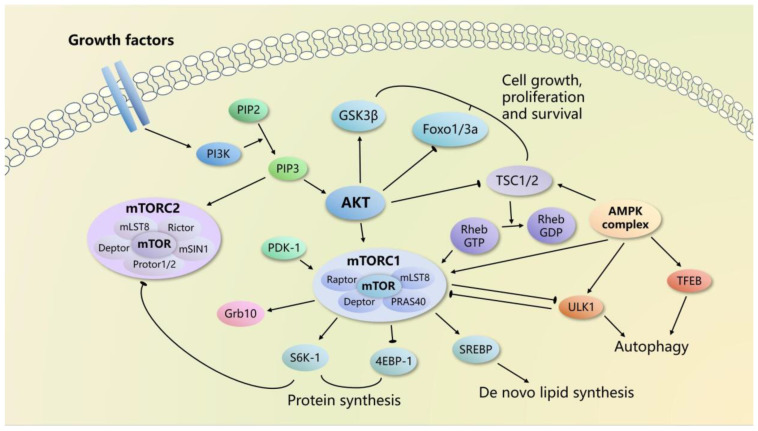
Overview of the mTOR signaling pathway and its associated regulators and functions.

mTOR exists in two structurally and functionally distinct complexes, known as mTOR complex 1 (mTORC1) and mTOR complex 2 (mTORC2) [12] (Figure 1). mTORC1 comprises five components: mTOR, mammalian lethal with sec-13 protein 8 (mLST8), regulatory-associated protein of mTOR (Raptor), DEP-domain containing mTOR-interacting protein (DEPTOR), and proline-rich AKT substrate of 40 kDa (PRAS40). mTORC2 is composed of six components: mTOR, mLST8, interacting protein 1 (mSIN1), rapamycin-insensitive companion of mTOR (Rictor), protein observed with Rictor 1 and 2 (Protor1/2), and DEPTOR [23,24,25,26]. For both mTORC1 and mTORC2, mLST8 acts as the positive regulator of mTOR activity while DEPTOR has a negative effect. For mTORC1, Raptor is a unique core subunit that modulates mTORC1 subcellular localization and substrate recognition by binding to the mTOR signaling motif, while PRAS40 inhibits mTOR activity [26,27,28]. Rictor is a key component of mTORC2 which is essential for mTORC2 function, while mSin1 and Protor1/2 serve as the regulatory subunits [12].

### 2.2. mTORC1 Signaling

mTORC1 is a crucial integrator of intracellular and extracellular signals, coordinating various biological processes, including protein synthesis, lipid synthesis, and autophagy [10,29]. The regulation of mTORC1 is well understood. Multiple factors, such as oxygen, energy, stress, and various growth factors, have been identified as activators of mTORC1 signaling by inhibiting the tuberous sclerosis complex (TSC) [18,30]. The TSC complex and the small guanine-5′-triphosphatase (GTPase), known as RAS homolog enriched in the brain (Rheb), are the major components responsible for transducing upstream signal to mTORC1 [31]. Whereas Rheb binds and activates mTORC1 directly, the activation of the TSC complex converts the active GTP-loaded Rheb into its inactive form, thereby negatively regulating mTORC1 activity [32]. Amino acids represent another main upstream stimulator of mTORC1. The availability of amino acids triggers the conversion of Ras-related GTP-binding protein homolog (Rag) family GTPases into their active conformation, which subsequently translocates mTORC1 to the lysosome, where Rheb is anchored and actives mTORC1 [33,34,35,36].

mTORC1 targets a variety of downstream molecules, including p70S6 kinase 1 (S6K1), eukaryotic translation initiation factor 4E-binding protein 1 (4EBP1), sterol regulatory element binding protein (SREBP) and Unc-51-like kinase 1 (ULK1). The promotion of protein synthesis by mTORC1 activation primarily relies on S6K1 and 4EBP1 [37]. mTORC1 phosphorylates S6K1 on its hydrophobic motif and 4EBP1 at multiple sites to enhance the translation efficiency of spliced mRNAs, which further modulates protein synthesis [38,39,40,41]. mTORC1 also regulates de novo lipid synthesis by activating SREBP through both an S6K1-dependent manner and the phosphorylation of an additional substrate, known as Lipin1 [42,43,44]. In addition, mTORC1 inhibits autophagy by blocking ULK1 activation by 5′ adenosine monophosphate-activated protein kinase (AMPK) and phosphorylation of transcription factor EB (TFEB) which regulates gene expression related to lysosomal biogenesis and autophagy [45,46,47,48].

### 2.3. mTORC2 Signaling

In contrast to the varied upstream signals of mTORC1, the activity of mTORC2 is mainly regulated by growth factors, including insulin, which signals phosphoinositide 3-kinase (PI3K) [49]. The inhibition of mTORC2 catalytic activity by the mSin1 PH domain is relieved upon binding to phosphatidylinositol-3,4,5-triphosphate (PIP3) generated by insulin/PI3K signaling [50]. The presence of insulin also facilitates the association of mTORC2 with ribosomes to enhance mTORC2 activity [51]. Besides, the negative feedback loop between insulin/PI3K signaling and mTORC1 also has a regulatory effect on mTORC2 activity. The phosphorylation of two mTORC1 downstream targets, S6K1 and growth factor receptor-bound protein 10 (Grb10), negatively regulates the insulin signal, thereby inhibiting mTORC2 activity [52,53].

AKT, a crucial effector of insulin/PI3K signaling, acts as the primary downstream target of mTORC2 [54]. Upon phosphorylation and activation, AKT modulates downstream substrates such as Forkhead box O1/3 (FOXO1/3a), glycogen synthase kinase 3 β (GSK3β), and TSC2 to promote cell growth, proliferation, and survival [55,56]. Moreover, mTORC2 regulates ion transport and cell survival through serum/glucocorticoid-regulated kinase 1 (SGK1) [54,57].

## 3. mTOR in Bone Metabolism

**Figure 2:**
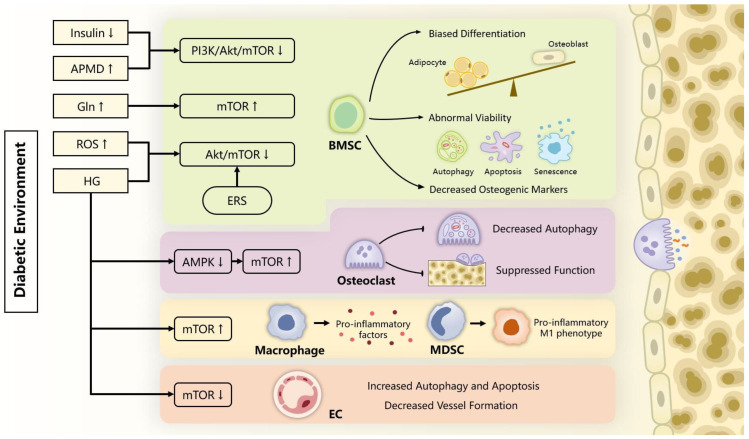
Implication of mTOR signaling in diabetic bone complications. Diabetes bone complications are characterized by imbalance between bone formation and resorption as well as immune and vascular alteration. This figure depicts how mTOR signaling is involved in these cellular abnormalities and contributes to the destructive changes in the diabetic skeletal system. The arrows in the yellow boxes indicate that the levels of the corresponding compounds rise or fall in the diabetic environment.

Bone metabolism is a complicated process requiring constant bone formation and resorption, which is regulated by the dynamic interplay between osteoblasts, osteoclasts, and various signaling pathways [58]. Exposure to a diabetic environment has profound adverse effects on bone metabolism, which compromises the structural and functional integrity of the skeletal system and leads to various clinical manifestations. The molecular process behind bone pathogenesis in hyperglycemia has been extensively studied, with particular attention given to mTOR signaling in recent years. In this subsection, we presented a summary of the current knowledge regarding the involvement of mTOR signaling in bone metabolism under high glucose conditions (Figure 2).

### 3.1. mTOR in BMSCs Osteogenesis and Bone Formation

BMSCs represent a multipotent precursor population, playing an essential role in bone homeostasis and formation via their osteogenic differentiation potential. The commitment of BMSCs to either an osteogenic or adipogenic lineage is delicately orchestrated by multiple mechanisms in physiological conditions. Nevertheless, mounting evidence suggests that the shift in BMSC differentiation toward adipogenesis in hyperglycemic conditions is likely associated with altered mTOR signaling activity.

The major pathophysiology of T1DM and T2DM involves insulin deficiency and insulin resistance, respectively. However, insulin and insulin-like growth factor (IGF-1) have been shown to exert bone anabolic effects through modulating mTOR activity [59]. Specifically, treatment with IGF-1 has been found to enhance the osteogenic differentiation of stem cells by significantly increasing the phosphorylation of AKT and p70S6K in a dose-dependent manner [20,59]. This effect was found to be reversed by inhibitors of PI3K and mTOR, such as LY294002 and rapamycin [20]. Furthermore, excessive activation of mTOR induced by the knockdown of *TSC2* inhibited insulin sensitivity in osteoblasts, leading to decreased osteogenic markers [60]. These results demonstrate the significant regulatory role of insulin and IGF-1 via the mTOR pathway in bone formation, which may be compromised in diabetic conditions due to reduced insulin stimulation. Indeed, extracts from Rehmanniae Radix Praeparata have been shown to alleviate bone loss and architectural deterioration in diabetic rats by promoting IGF-1 expression and activation of the downstream PI3K/AKT/mTOR pathway [61]. Glutamine, the richest semi-essential amino acid in the human body, signals to mTOR through Rag GTPase-independent mechanisms [62]. Under high glucose conditions, the increased glutamine concentration has been reported to hyperactivate mTORC1, which inhibited mTORC2 activity through the phosphorylation of S6K1, thereby reducing the runt-related transcription factor 2 (RUNX2) expression of murine mesenchymal stem cells (MSCs) along with impaired extracellular matrix calcification [22].

Either acute or chronic hyperglycemia leads to the accumulation of reactive oxygen species (ROS) [63], resulting in oxidative stress that can trigger cell senescence [64,65,66], apoptosis [64,67], and autophagy [65,68]. Such stress has been associated with the onset of DM and the development of diabetic bone complications [69]. As a critical regulator of BMSCs survival and function, the mTOR signaling pathway is involved in ROS-mediated pathological alterations. In osteoblastic MC3T3-E1 cells exposed to high glucose, ROS production significantly inhibited the AKT/mTOR pathway, upregulated autophagy-related genes, and boosted autophagy [68]. Despite its pro-survival role, mTOR-mediated autophagy appears to be a double-edged sword in diabetic impairment of osteogenesis. In hyperglycemia, the autophagy promoted by AMPK phosphorylation and mTOR inhibition partially rescued the compromised BMSCs osteogenic differentiation, and the downregulation of autophagy led to opposite outcomes [70]. Additionally, inhibition of mTOR by rapamycin in BMSCs treated with high glucose showed osteogenic protection and anti-apoptotic effects [65]. However, apoptotic BMSCs isolated from diabetic mice exhibited endoplasmic reticulum stress (ERS), which enhanced autophagy by inhibiting mTOR and induced apoptosis [67]. Besides, excessive ERS caused by glutathione peroxidase 7 (*GPx7*) knockdown negatively regulated AKT/mTOR activity and inhibited osteogenic differentiation of BMSCs [71]. Similarly, arginine pyrimidine (APMD), a core product of DM, downregulated the PI3K/AKT/mTOR pathway in periodontal cells and activated autophagy, thus promoting periodontal bone destruction [72].

Peroxisome proliferator-activated receptor γ (PPARγ) is an essential nuclear transcription factor for the balance between adipogenesis and osteogenesis of BMSCs. Recent studies have recognized the existence of crosstalk between mTOR and PPARγ during BMSC differentiation. The overexpression of miRNA188 in BMSCs directly inhibited Rictor, thus promoting the activity of PPARγ and enhancing adipogenic differentiation and adipose accumulation within the bone marrow microenvironment [73]. Furthermore, the heightened expression levels of DEPTOR in BMSCs isolated from osteoporotic mice induced by ovariectomy inhibited the nuclear translocation of transcriptional coactivator with a PDZ-binding motif (TAZ) thereby repressing RUNX2 transcription by facilitating PPARγ transcription [74]. These findings highlight the potential involvement of PPARγ in the mTOR-mediated osteogenesis in hyperglycemia, which requires further research for comprehensive elucidation.

Exposure to a hyperglycemic environment has been demonstrated to modify the characteristics of BMSCs and induce senescence [75] with a limited understanding of the contributions of the mTOR pathway [76]. Recent research indicates that reduced mTOR activity plays a critical role in the initiation of BMSCs senescence [77]. Interestingly, although BMSCs senescence stemming from autophagy under high glucose conditions is not affected by further mTOR inhibition using rapamycin [65], activation of the PI3K/AKT/mTOR pathway contributes to BMSCs senescence by inhibiting the Indian hedgehog pathway, and mTOR inhibitors reversed the senescent state of BMSCs [78]. Consistent with this, rapamycin-mediated mTOR inhibition protected BMSCs against oxidative stress-induced senescence, as evidenced by the mitigated senescence phenotype and mitochondrial damage [79].

### 3.2. mTOR in Osteoclast Formation and Bone Resorption

Osteoclasts are essential for bone resorption as they secrete degradative enzymes and create an acidic environment on the bone surface to demineralize bone tissue [80]. While some studies suggest that a diabetic environment may increase osteoclast differentiation and activity, ultimately leading to reduced bone mass and osteoporosis [81,82,83,84], others indicate that hyperglycemia and advanced glycation end products (AGEs) may inhibit osteoclast activity, with abnormal bone resorption and turnover [85,86,87].

The role of mTOR in regulating osteoclast differentiation and function is complicated [88,89]. The receptor activator of NF-kB ligand/osteoprotegerin/receptor activator of the NF-kB (RANKL/OPG/RANK) system is pivotal for osteoclastogenesis, with RANKL stimulating osteoclast differentiation by binding to RANK receptors on osteoclast precursor cells, while OPG acts as a competitive inhibitor [80]. mTORC1 has been shown to exert a biphasic regulatory effect on RANKL-directed osteoclastogenesis [90,91], with its activity in bone marrow-derived macrophages being activated in the early stages, but inhibited in the later stages following RANKL treatment [92]. Either inhibition of mTORC1 at an early stage or activation at a late stage during osteoclastogenesis suppressed osteoclast differentiation and bone resorption [92]. In contrast, a further study found that inhibiting mTORC1 at the late stage could also enhance osteoclast formation by promoting phosphorylation of the nuclear factor of activated T cells 1 (NFATc1) [91]. OPG inhibits mTOR by suppressing the PI3K/AKT pathway or activating the AMPK pathway, inducing autophagy and diminishing osteoclast viability, differentiation, and bone resorption activity [93,94,95]. Of note, the activation of autophagy does not necessarily suppress osteoclastogenesis. Mounting evidence has indicated that autophagy may be positively correlated with osteoclastic activity [89,96].

The detailed mechanism underlying the regulation of mTOR in osteoclastic activity in hyperglycemia is not well understood. It has been found that hyperglycemia inhibited the AMPK/mTOR/ULK1 pathway, thereby suppressing autophagy and inhibiting the formation and function of osteoclasts [97], whereas activation of the PI3K/AKT/mTOR pathway in diabetic mice reduced the RANKL/OPG ratio of osteoblastic cells, thus alleviating bone resorption [98]. Future studies are needed to obtain a better understanding regarding the involvement of the mTOR pathway in bone resorption in hyperglycemia.

### 3.3. mTOR in Inflammatory Response

DM is known to alter immune system components and has been considered an inflammatory disease [99]. The inflammatory microenvironment induced by hyperglycemia includes hyperactivated immune cells, increased chemokines and pro-inflammatory factors such as interleukin-1β (IL-1β) and tumor necrosis factor-α (TNF-α), and altered ratios of T helper 17 (Th17) cells and T regulatory (Treg) cells in the peripheral blood [100,101,102]. These factors exacerbate the suppression of osteoblastic activity [102,103]. mTOR is closely associated with immune and inflammatory responses. Inhibition of mTOR blocked NLR family pyrin domain containing (NLRP3) inflammasome activation and facilitated macrophage polarization toward the M2 subtype [104], which secretes multiple anti-inflammatory factors and promotes bone regeneration [105,106,107]. mTOR also promoted CD4+ T cell differentiation into Th17 cells, thus enhancing the adaptive immune responses [108]. In addition, targeting mTOR has been shown to regulate cytokine production and alleviate the development and severity of several inflammatory diseases, such as spondyloarthritis [109], rosacea [110], and acute gouty arthritis [111].

It has been shown that the high glucose-mediated mTOR phosphorylation in bone marrow-derived macrophages resulted in a senescent phenotype and upregulation of pro-inflammatory factors, ultimately exacerbating macrophage inflammation and periodontal bone destruction [112]. In addition, hyperglycemia was also demonstrated to promote macrophage pyroptosis and pro-inflammatory factor secretion by activating mTOR/4EBP1 and decreasing downstream ULK1 activity and autophagy flux, which in turn, aggravates alveolar bone resorption in periodontitis [113]. On the contrary, activation of the mTOR/AKT pathway inhibited pro-inflammatory factor production in hyperglycemic bone marrow-derived macrophages [114]. The conflicting results might be attributed to the different sources of macrophages. Moreover, the mTOR pathway regulates the responses of myeloid-derived suppressor cells (MDSCs), which restrict immune responses in bone repair [115]. High glucose promoted the differentiation of bone marrow-derived MDSCs into pro-inflammatory M1 macrophages, stimulating the accumulation of abnormal MDSCs in bone tissue [116]. This interference of immunosuppression depends on mTOR activation with phosphorylation of 4EBP1 and S6K1 and could be reversed by an mTOR kinase inhibitor [116]. Since M1 macrophage could inhibit osteoblastogenesis and facilitate apoptosis of MSCs [117,118], diabetes-stimulated M1 macrophage differentiation might be responsible for impaired bone defect regeneration in hyperglycemia. Hence, mTOR seems to positively regulate inflammatory responses in bone tissue and aggravate diabetes-induced bone metabolic aberrations.

### 3.4. mTOR in Bone Vascularity

The vascular components within the skeletal system play a vital role in regulating bone metabolism by facilitating the necessary supply of nutrients and biochemical factors, mobilizing bone progenitor cells, and balancing osteogenesis and osteolysis [119]. Diseased conditions such as DM have been shown to negatively impact the expression of angiogenesis-related genes in the skeletal system and impair BMSCs and pericyte functionality, leading to pro-angiogenic dysfunction [120,121,122]. T1DM mice demonstrated vascular lesions in bone, especially damage to type H blood vessels, which are responsible for coupling vascularization with osteogenesis [123], while the reduced microvascular blood flow in T2DM patients was notably linked to elevated cortical bone porosity [124]. Besides, inadequate vascularization under high-glucose conditions was detrimental to bone regeneration [125]. Overall, vascularization dysfunction constitutes a significant contributor to abnormal bone metabolism in diabetic conditions.

The mTOR pathway contributes to vascular dysfunction in diabetic bone by affecting endothelial cell (EC) functionality. mTOR activation is necessary for the tube formation of bone marrow-derived endothelial progenitor cells (EPCs) and human umbilical vein endothelial cells (HUVECs) [126,127], which can be inhibited in hyperglycemia through mTOR suppression. Mechanistically, the increased expression of circ-ADAM9 in EPCs induced by high glucose conditions negatively regulated its sponge microRNA-20a-5p, leading to increased autophagy and apoptosis in EPCs through inhibiting mTOR phosphorylation [128]. Additionally, the knockdown of circ-ADAM9 greatly reduced autophagy and apoptosis-associated protein expression in EPCs and increased tissue perfusion rates in diabetic mice [128]. Furthermore, upregulated miR-328 prevented the angiogenesis of HUVECs by suppressing AKT/mTOR pathway in a high-glucose low-serum environment [129], whereas activation of the PI3K/AKT/mTOR pathway effectively counteracts the anti-angiogenic effects of high glucose, promoting diabetic wound healing in vivo [130].

The function of the cellular components in the skeletal system under the diabetic environment is precisely regulated by the diabetic microenvironment (extracellular signals) and the consequent cascade of intracellular signals. Hyperglycemia is the most obvious and prominent extracellular signal. Its induced intracellular glucose metabolism disturbance and the ensuing redox imbalance cause oxidative stress and excessive production of ROS, which sets the cellular foundation for the establishment of skeletal complications [131,132]. At the molecular level, oxidative stress stimulates phosphatase activity, deactivating PI3K and disrupting the intracellular transduction of anabolic insulin signals [133]. Besides, ROS also results in the inactivation and degradation of AMPK by inhibiting its phosphorylation and promoting MG53-mediated ubiquitination [134]. The altered activity of PI3K and AMPK leads to the disruption of the normal intracellular transmission of mTOR signals. According to available findings, alterations in mTOR pathway activity mainly affect the downstream S6K1- and 4EBP1-mediated protein synthesis pathways and the ULK1-mediated autophagy pathway, which in turn leads to disruption of cellular component function and activity, thereby interrupting the balance between bone formation and bone resorption in the diabetic microenvironment.

## 4. Therapeutic Prospects

Strategies for alleviating the adverse impacts of hyperglycemia on bone metabolism are in urgent demand. The comprehensive regulatory effects of the mTOR pathway on the onset and development of diabetic bone complications render mTOR a potential therapeutic target. Here, we summarized several current mTOR-related drugs for combating skeletal deterioration (Table 1), providing insights for improving bone metabolism in the context of DM.

### 4.1. Inhibition of mTOR Pathway

The activation of AMPK inhibits mTOR, thus stimulating osteogenesis [149,150]. Metformin, a first-line agent for diabetic treatment, is known as an AMPK activator [151]. Multiple clinical studies have found that patients treated with metformin had lower fracture risk [152], and regulation of the AMPK/mTOR pathway might be one of the hidden mechanisms. By activating AMPK and inhibiting mTOR, metformin could regenerate the osteogenesis of ACSssuppressed by high glucose [135]. Besides, metformin-induced AMPK activation could inhibit the overactivation of mTORC1 caused by high glutamine in MSCs and eventually increase RUNX2 expression through the upregulated mTORC2/AKT-473 axis [22]. In addition to promoting osteogenesis, mTOR inhibition by metformin was also found to suppress adipogenesis mediated by PPAR-γ [136]. Interestingly, metformin might also indirectly activate mTOR in MSCs to contribute to osteogenesis. Shen et al. revealed that metformin could promote macrophage M2 polarization, and after coculture with metformin-pretreated M2 macrophages, MSCs exhibited higher PI3K/AKT/mTOR signaling activity and increased osteoblast differentiation and bone formation ability [153]. Despite its promotive effect on osteogenesis, it is concerning that the alteration in mTOR activity induced by metformin might result in the suppressed angiogenic function of BMSCs [154]. As for clinical practice, metformin is an insulin sensitizer with secondary protection against bone loss, thus metformin might not be considered an anti-osteoporotic drug [155]. The optimal dosing regimen for metformin to exert its bone repair function has not been fully understood either [156]. Studies showed that metformin promoted osteogenesis differentiation by regulating the AMPK pathway with a wide dose range from 0.5 μM to 500 μM [157].

Rapamycin, an mTORC1 inhibitor, might serve as a both beneficial and detrimental agent for DM and coexisting skeletal complications. For glucose metabolism, rapamycin administration is effective in promoting insulin secretion and glucose uptake in the short term [158], but chronic treatment may exacerbate hyperglycemia and insulin resistance [159]. In addition, the effects of rapamycin on osteoblastogenesis and osteoclastogenesis in vitro appeared to be dose-dependent, with lower doses promoting osteoblastogenesis and osteoclastogenesis while higher doses inhibited these processes [91,137,160,161]. In terms of osteoclastogenesis, a clinically relevant dose of rapamycin to treat cancer and suppress immune response was considered low by the above standard, thus clinically appropriate rapamycin might positively regulate osteoclastogenesis and facilitate bone resorption [91]. What is more, rapamycin might have negative effects on bone formation in young animals [138]. Therefore, the use of this particular drug in pediatric patients should be extra cautious [66].

However, studies have also found that inhibiting mTOR might lead to the inhibition of osteogenic differentiation. For example, Liraglutide, a glucagon-like peptide-1 receptor agonist (GLP-1RA) that could improve diabetic osteoporosis [162], was found to suppress the differentiation of osteoblasts by activating AMPK and inhibiting mTOR [139]. Interestingly, by activating the GLP-1 receptor and upstream PI3K/AKT pathway of mTOR, liraglutide could also promote osteogenic differentiation [163,164] and inhibit the apoptosis of osteoblasts [165] to regenerate bone formation. The activation of the two signaling pathways might result from different dosing regimens, and the precise function of mTOR in liraglutide treatment remains a challenge to be solved.

### 4.2. Activation of mTOR Pathway

In another way, the activation of PI3K/AKT/mTOR signaling might be beneficial for diabetic patients with bone complications. The activated PI3K/AKT/mTOR signaling pathway has been found to promote proliferation and differentiation [166]. Thus, developing a therapeutic approach to activate this signaling to maintain bone metabolism might be a worthy pursuit. As expected, Rehmannia glutinosa Libosch extracts were found to stimulate PI3K/AKT/mTOR signaling pathway and thus increase the proliferation and differentiation of osteoblastic MC3T3-E1 cells injured by high glucose [61]. In addition, activating upstream PI3K/AKT of mTOR, S-Equol and the combination of exendin-4 (Ex-4) and eldecalcitol (ED-71) could improve diabetic osteoporosis in vivo [98,167]. Furthermore, many other compounds that activate this signaling pathway exerted positive effects on osteoblasts, though lacking evidence proving their effects in the context of diabetes. For example, the activation of the PI3K/AKT/mTOR pathway by Naringin promoted the proliferation and differentiation of osteoblasts [143]. Moreover, activation of the PI3K/AKT/mTOR signaling pathway by tocopherol might cause attenuated ferroptosis in BMSCs and protect the cells from oxidative stress [140]. However, the effects of activating PI3K/AKT/mTOR signaling might be negative in osteoclasts. For example, the activation of the PI3K/AKT/mTOR signaling pathway by cholesterol could inhibit autophagy during osteoclast differentiation, thereby worsening osteoporosis [168].

Given the core position of the mTOR pathway in metabolic regulation and bone turnover, mTOR-targeted therapeutic strategies have the potential to combat diabetes-related bone diseases. In preclinical investigations, the dominant state of mTOR (activation or suppression) and the interaction with other signaling pathways need to be further clarified to better understand the pathology of diabetic bone complications. Besides, since mTOR exerts different impacts on different kinds of cells, how to target the desired cell types precisely is significant as well, to develop a much more precise treatment modality while reducing the side effects. Moreover, whether an mTOR-targeted agent adheres to a certain diabetes state needs to be elucidated to avoid misuse. Current clinical trials have mainly focused on alleviating diabetes and its complications with mTOR inhibitors, especially metformin. However, despite the potential osteogenic effects in preclinical investigations, metformin treatment could not yield optimal outcomes in improving bone metabolism of patients with T2DM, with BMD, trabecular bone score (TBS), and bone turnover marker levels taken into consideration [169,170,171]. Therefore, based on sufficient preclinical evidence, more clinical studies need to be conducted to evaluate the effectiveness of mTOR-targeted agents against diabetic bone diseases. For safety control, routine monitoring of glycemia and cardiovascular and renal function is required. For efficacy measurement, both glycemic and skeletal outcomes should be evaluated, and bone turnover-related parameters should be as thorough as possible, including BMD, TBS, markers representing bone formation and resorption, and assessment of fracture risk in the long term.

## 5. Conclusions

The mTOR signaling pathway is essential in regulating multiple aspects of skeletal development and homeostasis. The regulatory effects of mTORC1 on bone formation and resorption have long been recognized, and a growing body of evidence demonstrates that mTORC2 is also pivotal for bone physiology. Because of its crucial role in bone metabolism, the dysregulation of mTOR signaling in hyperglycemia is associated with the bone complications of individuals with DM.

We have thoroughly reviewed the current knowledge regarding the involvement of mTOR signaling in bone metabolism in hyperglycemia, including its effects on bone formation, bone resorption, inflammatory responses and bone vascularity. Despite these significant progresses, numerous challenges remain in elucidating the precise role of mTOR in diabetic bone complications and developing targeted therapeutic strategies. Specifically, it is crucial to further investigate the mechanisms by which hyperglycemia disrupts the integration of extracellular and intracellular signals and how this dysregulation affects mTOR pathway activity. Identifying the downstream effectors of mTOR that mediate bone metabolism in hyperglycemia is also a key priority. In addition, given the complex interplay between mTOR signaling and various physiological processes, developing tissue-specific approaches to modulate mTOR pathway activity and investigating potential side effects in non-skeletal organs will be critical for developing an effective therapy for diabetic bone complications.

## Figures and Tables

**Table 1:** Potential mTOR related agents in diabetes bone complications treatment.

Agents	Target Cell/Tissue (Environment)	Target	Main Finding	Ref.
Inhibition of mTOR				
Metformin	adipose-derived stem cells (ASCs) (40 mM high glucose culture environment)	mTOR	0.1 mM metformin reversed the osteogenesis inhibition of ASCs caused by high glucose via inhibiting mTOR and upregulating autophagy	[135]
	C3H10T1/2 MSCs (10% FCS plus either IID or PIO) (10% FCS plus AGD)	mTOR/p70	Metformin increased RUNX2 expression and inhibited PPARγ activity in MSCs through the suppression of the mTOR/p70S6K signaling pathway, thereby decreasing adipogenesis	[136]
	C3H10T1/2 MSCs (high-glucose conditions with glutamine)	AMPK/mTOR	Activation of AMPK by metformin inhibited high glutamine-induced mTORC1 hyperactivation and rescues RUNX2 through the mTORC2/AKT-473 axis	[22]
Rapamycin	human embryonic stem cells (hESCs) (mouse embryonic fibroblast-conditioned medium/serum-free medium)	mTOR	Rapamycin functioned as a potent stimulator of osteoblastic differentiation of hESCs by modulating mTOR and BMP/Smad signaling	[137]
	Five-week-old New Zealand White rabbits	mTOR	Direct infusion of rapamycin into proximal tibial growth plates decreased the size of the growth plate and inhibited overall long bone growth	[138]
PPARβ/δ Agonist	rat BMSCs (high glucose environment) SD rats (1% streptozotocin injected)	AMPK/mTOR	PPARβ/δ agonist promoted osteogenic differentiation of rat BMSCs through activating AMPK/mTOR-regulated autophagy and improved bone regeneration in type 1 diabetic rats.	[70]
Liraglutide	MC3T3-E1 (DMEM medium)	AMPK/mTOR	Liraglutide reduced the differentiation of MC3T3-E1 osteoblasts by regulating AMPK/mTOR pathway	[139]
Activation of mTOR signaling				
Rehmannia glutinosa Libosch Extracts	MC3T3-E1 (high glucose α-MEM medium) Wistar rats (high-fat diet and streptozotocin injection)	IGF-1/ PI3K/mTOR	The extracts increased the proliferation and differentiation of osteoblastic MC3T3-E1 cells injured by high glucose by activating the IGF-1/PI3K/mTOR pathway. Rehmanniae Radix Praeparata could prevent bone loss in type 2 diabetic rats.	[61]
Tocopherol	rat BMSCs (treated with H_2_O_2_)	PI3K/AKT/ mTOR	Tocopherol protected rat BMSCs from oxidative stress damage by activating PI3K/AKT/mTOR pathway	[140]
Pulsed Electromagnetic Fields (PEMFs)	MSCs (treated with 0.1 mg/mL of TNFα)	mTOR	PEMF increased the expression of osteogenic markers and promoted osteogenic differentiation of MSCs under TNF-α-mediated inflammatory conditions via mTOR activation	[141]
BMP-2	BMSCs (α-MEM medium)	mTOR	BMP-2 activated mTOR signaling pathway and downstream genes regulating protein anabolism to induce osteoblast differentiation	[142]
Naringin	Osteoblasts cultured from the differentiated BMSCs (DMEM medium)	PI3K/AKT/ mTOR	Naringin promoted proliferation and differentiation of osteoblasts by activating PI3K/AKT/mTOR pathway	[143]
Orthosilicic Acid	MG-63 and U2-OS (DMEM medium)	PI3K/AKT/ mTOR	Orthosilicic acid promoted osteogenesis in vitro by activating PI3K/AKT/mTOR signaling pathway	[144]
Transforming growth factor beta 1 (TGF-β1)	hFOB 1.19 (DMEM medium)	PI3K/AKT/ mTOR/S6K1	TGF-β1 induced the survival, osteogenic differentiation and migration of human hFOB 1.19 osteoblasts by activating the PI3K/AKT/mTOR/S6K1 pathway	[145]
Rutin	Periodontal ligament stem cells (PDLSCs) (α-MEM medium)	PI3K/AKT/ mTOR	Rutin increased proliferation and osteogenic differentiation of PDLSCs through G protein-coupled receptor 30 (GPR30)-mediated PI3K/AKT/mTOR signal transduction	[146]
1α,25-Dihydroxyvitamin D3 (1,25D)	Wild type mice (high-fat diet and streptozotocin injection) Osteoblasts (high glucose environment)	PI3K/AKT/ FoxO1, Sesn3/AMPK/ mTORC1	1,25D could reverse dysfunctional bone metabolism in type 2 diabetic mice through attenuating autophagy, by activating PI3K/AKT signaling, inhibiting FoxO1 and Sesn3/AMPK, and upregulating mTORC1.	[147]
Betulin (BET)	hFOB 1.19 (osteogenic medium and basal medium)	mTOR	BET increased the expression level of osteogenic differentiation markers and promoted mineralization by activating mitogen-activated protein kinases (MAPKs) and mTOR	[148]

## Data Availability

Not applicable.
